# Integrating Ecosystem Services in Nature Conservation for Colombia

**DOI:** 10.1007/s00267-020-01301-9

**Published:** 2020-05-28

**Authors:** Aracely Burgos-Ayala, Amanda Jiménez-Aceituno, Daniel Rozas-Vásquez

**Affiliations:** 1 Social-Ecological Systems Laboratory, Department of Ecology, Universidad Autónoma de Madrid, C/Darwin 2, 28049 Madrid, Spain; 2 Fundación Universitaria Juan de Castellanos, Carrera 11, 11-44, Tunja, Colombia; 3 Stockholm Resilience Centre, Stockholm University, 10405 Stockholm, Sweden; 4 Laboratorio de Planificación Territorial, Facultad de Recursos Naturales, Departamento de Ciencias Ambientales, Universidad Católica de Temuco. Rudecindo Ortega, 02950 Temuco, Chile

**Keywords:** Environmental education, Conservation policies, Environmental management, Ecosystem services, Colombia

## Abstract

The ecosystem services (ES) approach has been introduced in environmental policies and management to serve as a link between nature and society. Communication, education, and participation actions (CEPA) have the potential to facilitate this link. In this research, we evaluated how CEPA have been implemented in biodiversity conservation projects that consider ES. We used content analysis to review 182 biodiversity conservation projects executed by 33 environmental authorities in Colombia. We also used multiple correspondence analysis and cluster analysis to classify projects on the basis of the purpose of CEPA, type of CEPA, integration of CEPA, ES addressed, main stakeholders, and aim of conservation. We found that five aspects are key to fostering social engagement in environmental management projects: promoting explicit consideration of the ES approaches, increasing conservation efforts focused on the non-material benefits of the ES, integrating different types of CEPA, including overlooked key actors (e.g., indigenous communities and women), and developing and implementing social indicators. These considerations might lead environmental managers to revise their daily practices and, eventually, inform policies that foster an explicit link between CEPA and ES approaches.

## Introduction

Biological conservation is fundamental for sustainable development. The Convention on Biological Diversity (United Nations 1992) and the Conference of the Parties (COP 10, Nagoya 2010) highlighted the importance of promoting the public understanding of biodiversity conservation. For this purpose, they promoted the implementation of education and outreach programs to support the Strategic Plan for Biodiversity 2011–2020 (Conference of the Parties, in Nagoya, Japan, 2010) and the Aichi Targets (Decision IX/9). Therefore, communication, education, and participation actions (CEPA hereafter) were integrated and recognized as key tools and strategies for improving biodiversity conservation efforts (Hesselink et al. 2007; Jiménez et al. 2017; Wali et al. 2017). These CEPA consist of different social strategies (Ardoin and Heimlich 2013) that governments and practitioners use in conservation education and outreach programs (Salafsky et al. 2008; Jiménez-Aceituno et al. 2014, 2015).

Additionally, as a response to the United Nations’ Agenda 21 (1992), several environmental institutions and policies have been developed around the world to support the conservation and management of nature concurrent with human activities. At the country level, in most cases, conservation efforts have focused on the creation of protected areas (UNEP-WCMC-IUCN 2016). However, protected areas insufficient to maintain biodiversity and, furthermore, creation of protected areas promotes a disconnection between nature and humans (Gallopín 1991; Folke et al. 2011). Therefore, biological conservation is more likely to succeed when it focuses not only on biodiversity loss but on social-ecological systems, and the supply of ecosystem services (ES) (Berkes and Folke 1998; Mace 2014; van Oudenhoven et al. 2018). Biodiversity, i.e., the variety of living organism at all levels of organization on earth, influences the structures and functions of ecosystems, which might determine their capacity to deliver a range of ecosystem services to support human needs (Maes et al. 2013). Thus, the ES approach explicitly recognizes that society use and cares for nature when this in turn provides material and non-material goods and benefits for well-being and development (MEA 2005; Rozas-Vásquez et al. 2019). On the other hand, the ES approach is considered a potential means to better understand the relationship between nature and society. Some studies in Latin America have addressed that relation (e.g., Rozas-Vásquez et al. 2017; Bidegain et al. 2019; Cerda and Bidegain 2019). In 2005, The Millennium Ecosystem Assessment became a milestone for understanding the value of including ES in the conservation debate and the development of environmental policies (MEA 2005). This approach increasingly has been endorsed by policymakers at the global level, establishing a new conservation paradigm with greater possibilities to reconnect humans with nature (Mace 2014; van Oudenhoven et al. 2018).

The implementation of this new conservation paradigm requires a significant increase in social awareness and engagement (Hesselink et al. 2007; Hossain et al. 2018). CEPA are considered adequate and necessary to address the human-nature reconnection in social-ecological systems (Kramer et al. 2017; Hossain et al. 2018). There is evidence linking CEPA and ES. For instance, communication has been used to increase the understanding and awareness of decision makers about the ES approach (Hauck et al. 2013; Klein and Celio 2015). Education affects perceptions of cultural ES (Mocior and Kruse 2016; Ruppert and Duncan 2017). Participation has been studied in specific case studies related to payments for environmental services and spatial planning (Kosoy et al. 2008; Balderas et al. 2013; Dick et al. 2018). However, despite a range of examples, research about the CEPA of the conservation strategies in relation to ES is relatively sparse (Sodhi et al. 2010; Ruppert and Duncan 2017), and there is still a need to explore how CEPA are implemented in environmental management strategies that include ES (Klein and Celio 2015; Legagneux et al. 2018).

The objective of this study is to explore how biodiversity conservation strategies that include ES approach have integrated different CEPA to engage with society. We analyzed a sample of biodiversity conservation projects implemented in Colombia from 2004–2015 to determine how the projects implicitly or explicitly address different classes of ES, evaluate how CEPA have been used and incorporated into the projects, and classify projects to illustrate the different approaches to address environmental management challenges. This work is novel because it integrates ES and CEPA to explore the use of a variety of tools for social engagement that have been incorporated into biodiversity conservation strategies.

## Methods

### Study Area

**Fig. 1:**
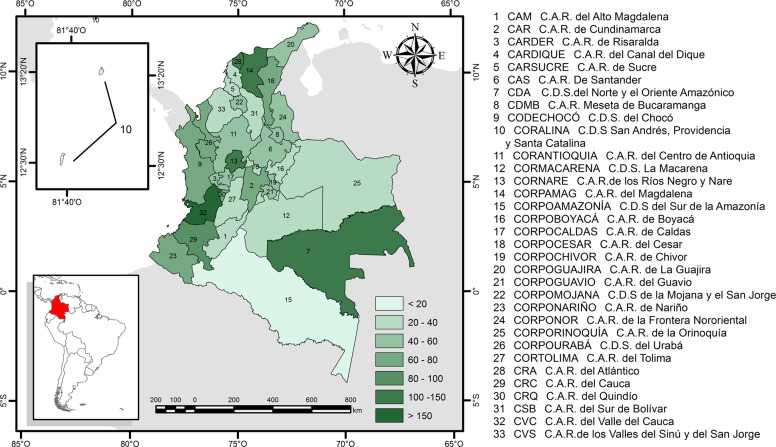
Location and number of projects developed by the Regional Autonomous Corporations (RCAs) in Colombia between 2004–2015. Acronyms in Spanish are shown in the legend: C.A.R. Corporación Autónoma Regional, C.D.S. Corporación Autónoma Regional de Desarrollo Sostenible

Colombia is located in South America, bounded by the Pacific Ocean, the Caribbean Sea, and five countries (Fig. 1). It extends over more than 2 million km² of five natural regions (Caribbean, Pacific, Andes, Amazon, and Orinoquia) (IAvH 2018). Colombia has high species richness and contains more than 10% of the world’s known species (Myers et al. 2000; Rangel-Ch 2005; Andrade-C 2011).

In 1991 Colombia enacted a new constitution, which adopted the concept of sustainable development and defined all of the current governmental environmental institutions in the country. Law 99 (MMA 1993) created the Ministry of Environment and the National Environmental System (NES). NES is in charge of setting guidelines, standards, activities, resources, and environmental programs and projects related to biodiversity management and conservation. The practical implementation of NES is carried out by 26 Regional Autonomous Corporations and seven in Sustainable Development (namely RACs hereafter; Fig. 1). We use the RACs as our spatial units of analysis for this study. RACs are delineated on the basis of territorial components such as geographic limits, type of ecosystems, geopolitical attributes, and biogeographic and hydro-geographic units (MMA 1994).

The RACs have the tasks of environmental management and implementation of a range of plans and projects related to sustainability, including education (MMA 1993; Canal and Rodríguez 2008; Rudas 2008). About 75% of the national environmental budget is transferred to the RACs. However, this funding represents 15% of the total budget managed by the RACS, which also generate money through activities such as collecting property taxes, rents, and payments for environmental services (Blackman et al. 2005; Sánchez-Triana et al. 2007; CGR 2017). Therefore, the RACs have both administrative and financial autonomy (MMA 1994).

The first National Biodiversity Policy (MMA 1996) was launched in 1996 and guides all environmental management decisions made by the RACs. It also entails an explicit integration of CEPA and ES in Colombian environmental policy. CEPA and ES are further developed in three key documents used by the RACs: the Policy of Social Participation in Conservation (MMA 2001), which is applied only in protected areas; the National Environmental Education Policy (MEN 2002); and the National Policy for the Integral Management of Biodiversity and its Ecosystem Services (NPIMBES) (MAVDT 2012). CEPA and ES are also integrated with the Colombian Biodiversity Action Plan (2016–2030) (MADS 2017); however, CEPA and ES have not been operationalized as environmental policy indicators (CGR 2012; Guhl and Leyva 2015; Muñoz-Montilla and Páramo-Bernal 2018).

### Data Collection

We searched for the total number of projects indicated by the annual reports of the 33 RACs for the period 2004–2015. Each RAC is regulated by a Regional Environmental Management Plan that is implemented through a Triennial Action Plan (TAP) (MAVDT 2004). Currently, three TAPs are available: 2004–2006, 2007–2011 and 2012–2015. The TAPs compile the projects executed in a given RAC and period. Since 2004 the RACs have released an annual report with information about these executed projects to be evaluated by the Colombian Ministry of Environment. When available, we downloaded the annual reports from the website of each RAC. We contacted the RACs and the Ministry of Environment to request the remaining documents. We obtained 322 reports out of an expected number of 396. The missing reports are mostly from the first TAP. From this set of reports, we generated a database of 2612 projects.

### Sampling Design

We took three steps to sample from the 2612 projects. First, we excluded those projects not directly related to biodiversity conservation, e.g., corporate visibility, internal control, administration, and document management and performance (*n* = 2212). Second, we conducted stratified probabilistic sampling. This technique is used to select a sample in each stratum independently, maintaining the representation of the population in each stratum (Thompson 2012). The total sample size was determined using the equation in Appendix 1 (*n* = 282), and we used TAP and RAC as strata from which randomly select the projects to be included in the sample (see Appendix 1). Third, we selected projects that explicitly or implicitly considered ES (Goldman and Tallis 2009; Hansen et al. 2015; Nordin et al. 2017) (*n* = 182; Table 1; Appendix 2, 3).

**Table 1:**
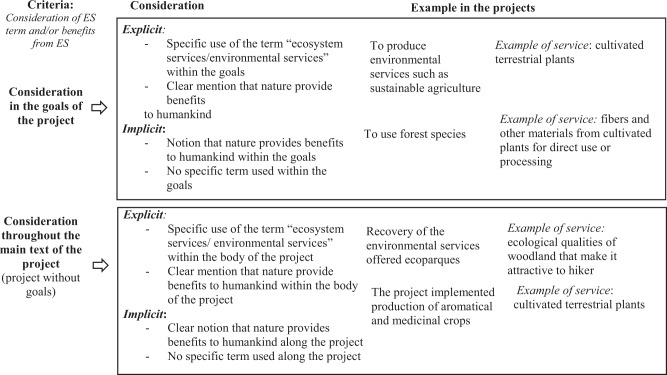
Set of criteria for determining the explicit or implicit consideration of the ES approach within the projects

### Content Analysis

We used content analysis to examine the sample of 182 projects. Content analysis is a systematic and repeatable examination of text to identify patterns and draw inferences about their meaning (Krippendorff 2004; Riffe et al. 2005). It allows one to reduce disparate and abundant information to a manageable amount of data without losing the original meaning (Riffe et al. 2005; White and Marsh 2006; Kohlbacher 2006). Content analysis requires defined sampling units, data collection units, and data units of analysis (Stemler 2001; White and Marsh 2006). Our sampling units were the 182 projects. The data collection units were phrases, tables, pictures, or photographs containing information related to specific characteristics of the projects, with an emphasis on their social characteristics. The data units of analysis were 52 items coded within 19 variables (Appendix 3).

We included five criteria in the coding process.

The first criterion was the type of ES addressed by each project (Table 1; variable 2, Appendix 3) To this end we used the Common International Classification of Ecosystem Services (CICES) proposed by Haines-Young and Potschin (2018), which has been implemented by the Intergovernmental Science-Policy Platform on Biodiversity and Ecosystem Services (IPBES 2012). CICES considers three main types of ES at section level: provisioning (e.g., timber, harvest, meat, fibers), regulating and maintenance (e.g., control of erosion rates, hydrological cycle and water flow regulation), and cultural (e.g., ecotourism, research, and education).

Second, we evaluated how the project applied CEPA (variables 3–7, Appendix 3). We assessed CEPA in three ways for each project. First, we gauged whether CEPA was a strategy for achieving an explicit project goal, or a goal in and of itself. Second, we assessed which types of CEPA were implemented (environmental communication, education, or participation). Third, we estimated the extent to which the components of the CEPA were related (Hesselink et al. 2007; Jiménez-Aceituno et al. 2014, 2015).

Third, we identified the main conservation actions (variable 8, Appendix 3). According to Salafsky et al. (2008) conservation actions are strategies aimed at land and water management (e.g., maintenance and recovery of parks and nature trails or soil protection and restoration), species management, raising awareness and education, or ensuring sustainable livelihoods (e.g., fostering sustainable rural production systems, bio-commerce, green markets).

Fourth, we identified the main stakeholders targeted by each project and the whether indigenous people, people of African descent, and women were explicitly engaged (variable 9–11, Appendix 3).

Fifth, we collected contextual variables for each project (variables 12 to 18; Appendix 3), such as explicit goals, spatial scale, TAP, budget, and duration of the project. We checked each project against the 52 items. Full details about each of the variables are in Appendix 3.

### Data Processing

We first used descriptive statistics to characterize 14 project variables: class of ES, whether ESs were considered implicitly or explicitly, the purpose of CEPA, main conservation actions, main stakeholders, whether women were targeted by the project, whether other underrepresented populations were targeted by the project, budget, duration, whether the project had explicit general goals and specific goals, TAP, continuity of the project, and number of projects by RAC (Appendix 3). To classify conservation projects on the basis of how they include society in their activities, we used XLSTAT 2015 software to conduct a multiple correspondence analysis (MCA) followed by a hierarchical cluster analysis (HCA). MCA is employed to analyze the associations between categorical variables, and provides quantitate variables and a standard measurement system for performing the HCA (Greenacre and Blasius 2006). We used Ward’s linkage method and Euclidean distances (Ward 1963) for the HCA. We applied the MCA and HCA to 11 variables (Appendix 3). We applied the HCA to the action coordinates of the main axes of the MCA and we used decreasing eigenvalues to select the axis with the greatest contribution to the variance (Bardat and Aubert 2007; García-Llorente et al. 2011; Jiménez-Aceituno et al. 2014, 2015). We used chi-square contingency-table tests to identify associations between each group of projects identified by the HCA and each of the analyzed variables.

## Results

### Main Characteristics of the Conservation Projects

We compiled 322 annual reports. The number of projects developed by a given RAC ranged from 1 to 33 (Fig. 1). General and specific goals were described in 64 and 16% of projects, respectively. About 37% of the projects were related to regulating ES, 34% to cultural ES, and 29% to provisioning ES. 48% of the projects were related to one class of ES, whereas 52% addressed two or three classes. Implicit consideration of ESs was more common than explicit consideration (35 and 12%, respectively).

Eight-seven percent of projects included CEPA, 18% as the main goal and 69% as a means to achieve other goals or a strategy for conservation.

Most of the projects targeted a range of stakeholders with their CEPA. Thirteen percent of the projects did not identify the main stakeholder. Local communities were the most commonly involved in projects and recognized as the main stakeholder (41%), followed by small-scale producers (18%), environmental leaders and groups (16%), decision makers and environmental practitioners (7%), and school children (5%). Indigenous and African-descended populations were the main stakeholder in 3% of the projects and targeted as a secondary stakeholder in 16% of the projects. Women were targeted in 2% of the projects as a secondary stakeholder.

The primary conservation actions developed by the projects were land and water management (51%). Sustainable livelihoods were the focus of 26% of the projects, whereas and awareness and species management were the targets of 19 and 4% of projects, respectively.

Twelve percent of project reports did not specify a budget. When budgets were provided, 77% corresponded to the implementation of CEPA (US $130 million), whereas the remaining 23% was spent on actions that did not directly target people. Most of the projects were implemented in TAP 2 (47%); 25 and 27% of projects, respectively, were implemented in TAP 1 and TAP 3. Twelve percent of projects continued during more than one TAP.

### Classification of Conservation Projects on the Basis of CEPA

The first two axes of the MCA explained 67% of the variance of the projects’ integration of social dimensions (see Appendix 4). The first component (F1; 50% of the total variance) represented the level of implementation of the CEPA actions in the projects. Positive scores reflected short projects that implemented any CEPA. Conversely, negative scores reflected projects where multiple CEPAs were implemented in an integrated way as a strategy for conservation. The second component (F2; 17% of the total variance) captured the purpose of the social component in the projects. Positive scores corresponded to projects that used participation actions to improve regulating ES in land and water management. Negative scores reflected projects that focused on cultural ES and aimed to promote society environmental awareness and education.

**Fig. 2:**
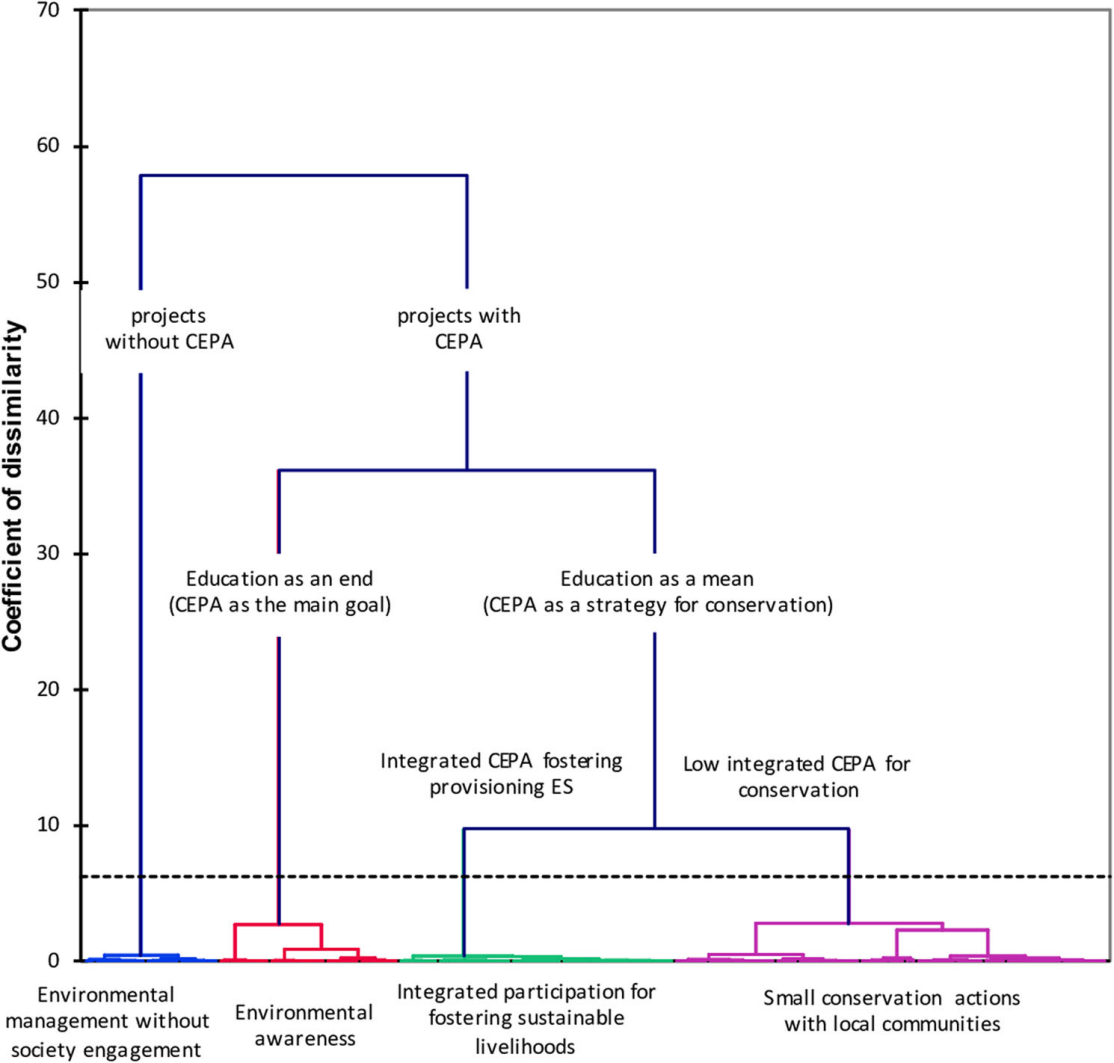
Hierarchical Cluster Analysis (HCA) of the projects developed by the Regional Autonomous Corporations in Colombia (2004–2015) based on how they include society

Application of HCA to the two first components of the MCA and revealed four types of projects (Appendix 5): environmental management without societal engagement, environmental awareness, integrated participation for promoting sustainable livelihoods, and small conservation actions with local communities (Fig. 2; Table 2).

**Table 2:** Description of the four types of conservation projects based on the ES sections, type, integration, and purpose of CEPA actions implemented (communication, education, participation), conservation action, stakeholders, spatial scale, budget, and duration

*Type of the project*	*Purpose of CEPA actions*	*ES Section*	*Type of CEPA actions*	*Integration of CEPA actions*	*Main Conservation action*	*Main stakeholder targeted*	*Spatial scale*	*Budget*	*Duration*	*Some examples of projects (RAC)*^*a*^
Environmental management society engagement (13%)	Without CEPA actions	Regulating^b^	Without CEPA actions	Without CEPA actions	Land and water management	No society involvement	–	–	1 year	1. Environmental recuperation and protection in the municipalities of the department of Atlántico (CAR) 2. Protection and restoration of eroded soils roads in The Saladito township (CVC)
Environmental awareness (18%)	CEPA as an end: the main goal	Cultural	Communication/ Education^b^	–	Education and awareness	Env. people/School children	Regional	–	–	1. Communication and environmental education (Corpourabá) 2. Environmental education for social training and community participation (Corpochivor)
Integrated participation for fostering sustainable livelihoods (27%)	CEPA as a mean: as a strategy for conservation	ProvisioningRegulating	Communication, Education and Participation	High	Livelihood/Land and water management^b^	Small-scale/Local communities^b^	–	High	2–4 years	1. Implementation of sustainable production alternatives and training to groups of fishermen of the jurisdiction (Corpomojana). 2. Recovery of degraded areas in the Peasant farmers reserve area (Reservas campesinas) (CDA)
Small conservation actions with local communities (42%)	CEPA as a mean: as a strategy for conservation	Regulating^b^	Low communication^b^/Low education^b^	Low and medium	Species management/Land and water management^b^	Local communities	Local	Low	2–4 years/ >4 years	1. Recovery and Sustainable Management of the Bitter Palm in indigenous areas (Carsucre). 2. Comprehensive environmental management articulated to local communities (Corantioquia)

^a^See Appendix 1

^b^High frequency in the group, although without statistical evidence

Environmental management without societal engagement accounted for the lowest percentage of projects (13%). These projects were usually implemented over one year and did not involve stakeholders. The main conservation action of this group was land and water.

The environmental awareness group included 18% of projects. Most focused on cultural ES and implementing educational actions. The group was characterized by use of public communication for environmental sustainability, and environmental leaders and groups and school children were the main stakeholders.

Integrated participation for promoting sustainable livelihoods included 27% of the projects. These projects were categorized as provisioning and regulating ES. They implemented different types of CEPA, being the three types of actions highly integrated in this group. The CEPA targeted small-scale producers, such as associations of farmers, fishermen, and artisans. Sustainable livelihoods and land and water management were the main conservation actions. Their duration was 2–4 years or more, and their budgets were high budget (>US$500,000).

The remaining projects (42%), characterized as small conservation actions with local communities, had small budgets. They implemented CEPA with low and medium integration relative to projects in other groups. These projects employed a variety of conservation actions, e.g., species management.

## Discussion

### Integrating the ES and CEPA Approaches in Environmental Management

**Fig. 3:**
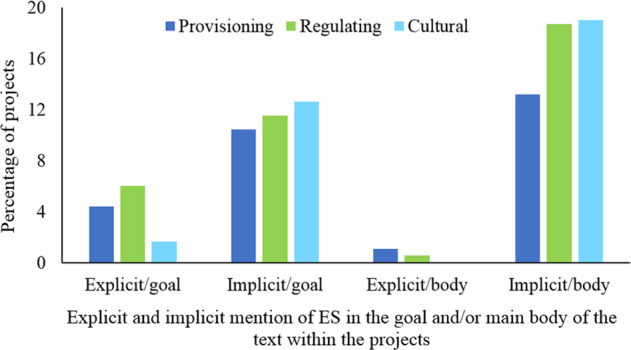
Percentage of explicit and implicit mention of ES sections (provisioning, regulating, cultural) within the goals or the main body of the text in the conservation projects

In 2005, The Millennium Ecosystem Assessment created momentum for the integration of ES in environmental conservation policies around the world (MEA 2005). In 2012, Colombia became one of the first countries to adopt the ES concept in its NPIMBES). This policy made the integration of the ES concept mandatory for the management of Colombia’s natural resources (MAVDT 2012). Our results indicate that the implementation of conservation management strategies has considered the three classes of ES defined by CICES. However, these ES rarely are mentioned explicitly in project reports (Fig. 3). This is considered a critical issue to be addressed by conservation management strategies and planning in order to take advantages of the ES approach, especially in terms of facilitaging communication,information and understanding from a range of actors and decision-makers (Rozas-Vásquez et al. 2019). In addition, explicit consideration of ES might enhance a strategic analysis of trade-offs for preventing bias in the management and consequent supply of certain ES (Spyra et al. 2019). Given that all the analyzed projects were approved before the NPIMBES, we could not determine whether the explicit mention of ES in this policy has contributed to an increase in the explicit mention and use of ES approaches by RACs during recent years. Furthermore, the implementation of ES approaches in environmental management will require carefully examination of the tools used for social engagement (Hesselink et al. 2007; van Oudenhoven et al. 2018). We classified conservation projects to explore how these tools (i.e., CEPA) are integrated into environmental management. To the best of our knowledge, this is the first assessment of the wide variety of educational tools used in environmental management practices that address ES. Our methods could be extended in other analyses of environmental management documents because they allow comparison of a series of implemented projects even when information on the projects is sparse and disorganized (Sodhi et al. 2010; Klein and Celio 2015; Ruppert and Duncan 2017). Classifications of projects could support governmental institutions in evaluating their management performance and monitoring their daily practices. However, the use of classifications poses some challenges. For example, the units of analysis in our content analysis mainly were based on theoretical concepts, which often are difficult to infer from the available data (Hsieh and Shannon 2005). Therefore, it was difficult, for example, to identify and differentiate among the ES classes defined by CICES, and to evaluate the implicit or explicit mention of these ES (see Table 1).

### Classification of Conservation Projects

Our analysis identified four types of conservation projects on the basis of how the different educational tools have been implemented and the different ES types considered. A relatively low percentage of projects did not include any tool for social engagement. These projects might reflect a focus on strict environmental protection rather than ES supply. Our approach is different from earlier classifications of conservation projects (e.g., Kapos et al. 2008; Salafsky et al. 2008) that considered education as a particular type of conservation action or that did not address integration among communication, education, and participation actions (Fien et al. 2001).

The percentage of projects that focused on environmental awareness was much smaller than reported by similar studies in other countries. For example, Jiménez-Aceituno et al. (2014) found that projects that raised awareness and understanding about biodiversity were the second most common conservation projects developed in Spain. In these projects the main targets were information disseminators, such as teachers. Similarly, the audiences targeted by the environmental awareness projects in our study do not have a direct impact on ecosystems and have limited influence on decision-making. Furthermore, decision makers were not the main targeted audience of any of the groups of projects identified by the cluster analysis. Conservation actions that do not increase the environmental awareness of these influential actors may limit the environmental decision cascade (Moreno et al. 2014).

Other projects use CEPA as a mean to achieve other conservation goals, i.e., developing CEPA is not the main goal of the project, but CEPA aimed to facilitate other conservation actions. Projects related to integrated participation for fostering sustainable livelihoods were characterized by their implementation of all types of CEPA, which contributed to improving the effectiveness of the projects (Fien et al. 2001; Hesselink et al. 2007; Zorrilla-Pujana and Rossi 2016). Furthermore, the long duration of projects in this group might foster the empowerment of local communities and small-scale producers, especially via participation. This group shares some characteristics with the “integrated participation” described by Jiménez-Aceituno et al. (2014). However, the former projects mainly aimed to provide instrumental training and build networks with other organizations, whereas our group focused on fostering sustainable livelihoods by generating economic benefits for local communities and small-scale producers. This difference might be related to the environmental management interests in countries of the global north and global south. The later countries, such as Colombia, have a stronger tradition of working with people whose economies have been historically and closely connected to biodiversity (Balvanera et al. 2012).

Projects that conducted small conservation actions with local communities generally did not integrate CEPA. One of the most common types of projects in this group was basin management plans in the diagnostic stage. A relatively new regulation (MADS 2014) demands broad public participation throughout these projects. Before this regulation, CEPA strategies primarily informing the users about the basin delineation (MMA 2002). Understanding this limited CEPA integration will require further exploring the relevance given to different types of CEPA strategies when managing biodiversity and ES.

### Stakeholders Addressed by the Conservation Projects

Our results revealed a wide range of stakeholders (e.g., local communities, children, decision-makers, indigenous) targeted by the management projects, mainly because of the scope and objectives of those projects. This is considered as a potential positive aspect because engagement of a variety of stakeholders is a key issue for an effective bidiversity conservation (Goldman et al. 2008; Kramer et al. 2017; Hossain et al. 2018). However, local communities were the most frequent stakeholders targeted by the projects. Conservation based on the community traditionally has been successful (Wali et al. 2017), and communities along with artisans, farmers, or fishermen are strategic stakeholders for sustainable development. These actors have a direct influence on the ecosystems, determining their conservation and the subsequent ES supply (Berkes et al. 2009; Bennett et al. 2019).

Children seldom were targeted by conservation projects. To conserve biodiversity and its ES in the future is necessary that children are aware of nature and the complex social-ecological relationships related to its conservation (Xiong et al. 2016). Decision-makers and government practitioners seldom were targeted by CEPA. These stakeholders have great ability to influence environmental decisions, but they need to be aware of the importance, values, and consequences of the use and management of ES to make informed decisions (Moreno et al. 2014; Beery et al. 2016; Ruppert and Duncan 2017). Another weakness we perceived was the lack of CEPA aimed at indigenous communities. In Colombia there are 696 indigenous reservations (resguardos in Spanish). A reservation is a collective property title of the land, with a legal basis that protects the territory and both the cultural and political autonomy (van der Hammen 2003). Twenty-eight percent of Colombia is indigenous territory (Mosquera et al. 2016). Indigenous communities are an invaluable source of knowledge of sustainable practices for ES supply and nature protection (Delgado-Serrano et al. 2017; Sangha et al. 2017; Wali et al. 2017). Because conservation requires intercultural dialogs to co-produce knowledge, indigenous communities need to be integrated into environmental policies and decisions (Pascual et al. 2017; Corrigan et al. 2018; Díaz et al. 2018). Women were not targeted as stakeholders in most of the projects, but women are central in global environmental agendas (Egaga and Akinwumi 2015; Yang et al. 2018). Women also teach and instill awareness on sustainability in young people, families, and communities (Wals and Kieft 2010; Mukoni 2015). Therefore, there is a great opportunity to foster the link already established among women, environmental education, and conservation, that can be reinforced by institutional environmental management strategies.

### The Lack of Social Indicators for Monitoring and Evaluation

Public education and awareness are persistent and key challenges for biodiversity conservation (Brown 2003; Kramer et al. 2017; Hossain et al. 2018). In 2020, education will be the focus for achieving the Aichi targets, particularly in awareness and integrated participation (goals A and E). We found that CEPA was prevalent in the conservation projects, which means that the RACs are trying to promote people’s environmental awareness, empowerment, and skills to support conservation (Sodhi et al. 2010; Moreno et al. 2014; Hutcheson et al. 2018). Different uses of CEPA (i.e., as a mean and as an end) support sustainability (Sterling 2010; Jiménez-Aceituno et al. 2014). However, our results indicate that few projects use CEPA as an end and, consequently, education rarely appears to be considered as a non-material benefit of ES (e.g., nature contributing to the learning process of the people targeted by the CEPA) in the environmental management practices of the RACs.

Non-material benefits of ES are rarely addressed in decision-making (Chan et al. 2012; Satz et al. 2013). Similarly, environmental management practices are mainly focused on achieving environmental indicators [in Colombia established in the General Environmental Law 99 (MMA 1993), and its Resolution 0964 (MAVDT 2007); e.g., increasing the number of hectares under environmental protection], whereas social indicators are not commonly included in the environmental legislation. Our results follow this trend, as CEPA is rarely considered as a non-material benefit of ES. This highlights the need to include a set of context-specific social indicators (besides social and economic measures) that are able to enhance the traditional solely environmental view for a more effective assessment of the real impacts of environmental management practices (Zorrilla-Pujana and Rossi 2016; Sterling et al. 2017; Dacks et al. 2019).

Additionally, our results indicate that there are substantial deficiencies in the generation, storage, and monitoring of the information related to environmental management activities (Newborne et al. 2010). Examples include the absence of the annual reports generated by the RACs, the difficulty of obtaining annual reports from environmental institutions, and the fact that many conservation projects do not document their goals or budgets. The establishment of indicators, information and evaluation systems, and monitoring strategies to provide inference to the achievements and failures of conservation practices is crucial for improving and promoting more consistent decision-making processes (Ferraro and Pattanayak 2006; Zorrilla-Pujana and Rossi 2016). Information about environmental management activities is particularly needed in Colombia and other highly diverse countries where environmental and education funding are quite limited, and rigorous analysis of environmental investments have been historically a pending task. Such information must be created, maintained, analyzed, and communicated to ensure that environmental investments secure the countries natural capital (Galán and Canal 2002; Waldron et al. 2013).

## Concluding Remarks

Finally, as a relevant contribution to the current knowledge, this research suggests five key issues to enhance environmental management practices and foster society engagement: (1) promote the explicit consideration of ES in policies, plans and programs in order to increase the advantages of using this approach as facilitator for communication, information and action between science, policy-makers and a range of stakeholders (Rozas-Vásquez et al. 2019), (2) increase conservation efforts focused on education, recognizing the non-material benefits of ES (Chan et al. 2012); (3) integrate multiple types of CEPA from the design to implementation stage of projects in order to increase the effectiveness (Fien et al. 2001; Zorrilla-Pujana and Rossi 2016); (4) include a range of actors focusing on diversity and inclusiveness (Egaga and Akinwumi 2015; Sangha et al. 2017), especially those who usually are not well represented in traditional environmental management strategies (e.g., indigenous communities and women); (5) develop and implement social indicators for environmental management that complement the more commonly used environmental indicators (Newborne et al. 2010; Dacks et al. 2019). We believe these considerations might support environmental managers to revise and improve their daily practices and, at the same time, contribute to the achievement of political mandates that foster social engagement for conserving biodiversity and its ES.

## Supplementary Information

Supplementary Material 1

Supplementary Material 2

Supplementary Material 3

Supplementary Material 4

Supplementary Material 5
