# Supplementary material for: Integrating Ecosystem Services in Nature Conservation for Colombia
Source: Environ Manage. 2020 May 28;66(2):149–61. doi: 10.1007/s00267-020-01301-9 (PMC7320067; doi:10.1007/s00267-020-01301-9)
Supplement: Supplementary file 1 — Supplementary Material 1 [file 267_2020_1301_MOESM1_ESM.pdf]

Appendix 1. Sample design and selection

Sample design:

We used the following equation to calculate the total sample size of conservation projects, which is adequate when the population is finite and known (N=2212) (Zar, 1996).

$$n = \frac{NZ_{\alpha}^{2}pq}{(N-1)d^{2} + Z_{\alpha}^{2}pq}$$

Where:

n = sample size
N = population size
Z = confidence level
p = probability of success
q = probability of failure (q=1-p)
d = margin of error

We considered a confidence level of 95% (Z=1.95), a probability of success of 70% (p=0.7), a probability of failure of 30% (q=0.3) and a margin of error of 5% (d=0.05). Probability of success refers to the probability of finding ES projects within the population of projects (N), and was based on Goldman & Tallis (2009.

Sample selection:
We used a stratified sampling to assure a representative sample of projects considering two stratum: TAPs (Triennial Actions Plans; n=3) and RACs (Regional Autonomous Corporations; n=33) (see table in the attached Appendix 1). We followed four steps:

First, we calculated the percentage of projects within each TAP in the whole set of projects: TAP1, 21%; TAP2, 45%; and TAP3, 34% . Then, for the 282 projects in the sample, we calculated the proportional number of projects within each TAP: TAP1 (n=58); TAP2 (n=125); and TAP3 (n=99). Second, we calculated the percentage of projects implemented by each of the 33 RACs within each TAP (e.g., the RAC Corpocesar implemented 7 projects within TAP1, i.e., 1,52% of the total number of projects implemented within that TAP), and the proportional number of projects in the sample. Fourth, to avoid bias on project selection, we marked each of the 2212 projects with a random number using Microsoft Excel (2015). Then we selected the projects with a higher number within each TAP and RAC number of projects -according to the required number as defined in the previous step).

RAC	TAP1			TAP2			TAP3			Total number of projects in the sample [projects in the population]
	Total projects	% Projects by TAP	Number of projects in the sample	Total projects	% Projects by TAP	Number of projects in the sample	Total projects	% Projects by TAP	Number of projects in the sample	
1. Corpocesar	7	1,52	**1**	25	2,53	**3**	34	4,46	**4**	**8** [66]
2. Corpoguajira	24	5,22	**3**	17	1,72	**2**	11	1,44	**1**	**6** [52]
3. Corponor	15	3,26	**2**	12	1,21	**2**	21	2,75	**2**	**6** [48]
4. CDMB	16	3,48	**2**	28	2,83	**4**	9	1,18	**1**	**7** [53]
5. CAS	11	2,39	**1**	15	1,52	**2**	21	2,75	**3**	**6** [47]
6. Corpoboyacá	8	1,74	**1**	15	1,52	**2**	10	1,31	**1**	**4** [33]
7. Corporinoquía	9	1,96	**1**	13	1,31	**2**	10	1,31	**1**	**4** [32]
8. Corpochivor	7	1,52	**1**	30	3,03	**4**	10	1,31	**1**	**6** [47]
9. Corpoguavio	27	5,87	**3**	20	2,02	**3**	5	0,66	**1**	**7** [52]
10 CAR	26	5,65	**3**	24	2,43	**3**	14	1,83	**2**	**8** [64]
11. Cormacarena	12	2,61	**1**	9	0,91	**1**	13	1,70	**2**	**4** [34]
12. CDA	10	2,17	**1**	44	4,45	**5**	61	7,99	**8**	**14** [115]
13. CAM	8	1,74	**1**	10	1,01	**1**	9	1,18	**1**	**3** [25]
14. Corpocaldas	12	2,61	**1**	14	1,42	**2**	17	2,23	**2**	**5** [43]
15. CRQ	15	3,26	**2**	21	2,12	**3**	28	3,67	**3**	**8** [64]
16. Carder	14	3,04	**2**	21	2,12	**3**	17	2,23	**2**	**7** [42]
17. Cortolima	0	0,00	**0**	7	0,71	**1**	29	3,80	**3**	**4** [36]
18. CRC	49	10,65	**5**	17	1,72	**2**	25	3,28	**3**	**10** [91]
19. Corponariño	37	8,04	**5**	20	2,02	**3**	15	1,97	**2**	**10** [72]
20. Corpourabá	30	6,52	**4**	16	1,62	**2**	32	4,19	**4**	**10** [78]
21. Corantioquia	14	3,04	**1**	14	1,42	**2**	20	2,62	**3**	**6** [48]
22. Codechocó	9	1,96	**1**	44	4,45	**6**	12	1,57	**2**	**9** [65]
23. CVC	69	15,00	**10**	184	18,60	**22**	119	15,60	**18**	**50** [374]

24. Corpamag	9	1,96	**1**	80	8,09	**10**	46	6,03	**6**	**17** [135]
25. Carsucre	3	0,65	**0**	22	2,22	**3**	11	1,44	**1**	**4** [36]
26. Corpoamazonía	0	0,00	**0**	8	0,81	**1**	6	0,79	**1**	**2** [14]
27. Cardique	1	0,22	**0**	17	1,72	**2**	10	1,31	**1**	**3** [28]
28. Coralina	2	0,43	**0**	6	0,61	**1**	8	1,05	**1**	**2** [16]
29. Cornare	0	0,00	**0**	91	9,20	**13**	44	5,77	**6**	**19** [135]
30. Corpomojana	14	3,04	**2**	34	3,44	**4**	8	1,05	**1**	**7** [56]
31. CRA	0	0,00	**0**	66	6,67	**8**	63	8,26	**8**	**16** [129]
32. CSB	0	0,00	**0**	32	3,24	**4**	5	0,66	**1**	**5** [37]
33. CVS	2	0,00	**0**	13	1,31	**2**	20	2,62	**3**	**5** [35]
**Total number of projects (%)**	460 (21%)		**58** (21%)	989 (45%)		**125** (45%)	763 (34%)		**99** (34%)	282 [2212]

References

Zar JH (1996) Biostatistical Analysis, 5th edn. Upper Saddle River, Prentice Hall, New Jersey
