# Supplementary material for: Integrating Ecosystem Services in Nature Conservation for Colombia
Source: Environ Manage. 2020 May 28;66(2):149–61. doi: 10.1007/s00267-020-01301-9 (PMC7320067; doi:10.1007/s00267-020-01301-9)
Supplement: Supplementary file 2 — Supplementary Material 2 [file 267_2020_1301_MOESM2_ESM.pdf]

Appendix 2 List of the 182 projects selected in the RACs

Title of the project	RAC implementing
Fortalecimiento de una cultura ambiental y de participación ciudadana	CADER
Incorporación de las determinantes ambientales en los instrumentos de planificación y el ordenamiento territorial	CADER
Fortalecimiento de la gestión del conocimiento	CADER
Consolidación de un circuito para el turismo sostenible en la cuenca media alta del rió Otún	CADER
Implementar bosques FLEGT - Colombia	CADER
Planificación y gestión ambiental local y regional	CADER
Apoyo a la comercialización de productos derivados del aprovechamiento sostenible de la biodiversidad	CADER
Fomento y apoyo al ecoturismo en torno al río Magdalena	CAM
Uso sostenible de la biodiversidad y negocios verdes	CAM
Planificación y gestión integral del recurso hídrico	CAM
Gestión y abastecimiento hídrico	CAR
Gestión de distritos de riego	CAR
Planeación de la ordenación y el manejo de cuencas hidrográficas	CAR
Conservación, recuperación y restauración de los ecosistemas y la oferta natural	CAR
Gestión proyectos ambientales	CARDIQUE
Ordenamiento y manejo integrado Ciénaga de la Virgen	CARDIQUE
Gestión de los recursos naturales	CARDIQUE
Estudio y recuperación de la caña guadua en la jurisdicción de Carsucre	CARSUCRE
Recuperación y manejo de la caña flecha en zonas indígenas	CARSUCRE
Recuperación y manejo sostenible de la palma amarga en zonas indígenas	CARSUCRE
Formación de promotores ambientales	CAS
Conocimiento y conservación de la biodiversidad	CAS
Fotalecimiento al control, seguimiento y monitoreo a los usos y provechamiento de los recursos naturales y a las obras de desarrollo	CDA
Apoyo al monitoreo e implementación de una estrategia integral y sostenible de reducción de cultivos ilícitos y promoción del desarrollo alternativo en Colombia, bajo la cultura de la legalidad 2014	CDA
Recuperación de áreas degradadas en la zona de reserva campesina año 2012, Departamento del Guaviare	CDA
Apoyo a los procesos organizativos para la producción y ventanilla verde de servicios como son ecoetiquetados y ecoturismo	CDA
Evaluación del estado de las poblaciones de quelonios del género *Podocnemis* en la cuenca baja del río Guaviare en el departamento de Guainía	CDA
Fortalecimiento al proceso de planificación ambiental, local, para la conservación, recuperación y uso sostenible del sistema de humedales del municipio de Mitú	CDA
Implementación de sistemas productivos sostenibles como estrategia de desarrollo regional de los departamentos de Guainía, Guaviare y Vaupés	CDA
Restauración de chagras tradicionales como alternativa para el retorno y ocupación permanente de los grupos Nukak desplazados por factores exógenos sector estratégico resguardo Nukak Maku departamento de Guaviare	CDA
Conservación, investigación y propagación de la flora del nororiente de Santander en el Jardín Botánico Eloy Valenzuela	CDMB
Educación ambiental y participación social para la gestión ambiental integrada y compartida en el área de jurisdicción de la CDMB	CDMB
Fomento y apoyo al sector productivo industrial en la implementación de la producción más limpia y el acceso a mercados verdes	CDMB
Restauración, conservación y uso sostenible de los humedales del Medio y Bajo Lebrija	CDMB
Conservación, investigación y propagación de la flora del nororiente de Santander en el Jardín Botánico Eloy Valenzuela	CDMB
Administración para la gobernanza forestal en el departamento del Chocó	CODECHOCÓ
Ordenamiento de los recursos naturales y del territorio en el departamento del Chocó	CODECHOCÓ
Implementación de estudio base para especies forestales amenazadas	CODECHOCÓ
Implementación de plantaciones forestales en comunidades indígenas, negras y campesinas del bajo atrato en el departamento del Chocó	CODECHOCÓ
Agroforestería comunitaria asociada a mercados verdes y biocomercio en el departamento del Chocó	CODECHOCÓ
Formulación e implementación del plan de ordenación y manejo de la cuenca del río Condoto e implementación de once planes adoptados por Codechoco	CODECHOCÓ
Fortalecimiento de la administración de áreas protegidas en la Reserva de Biosfera Seaflower	CORALINA

Ordenación y aprovechamiento de los bosques naturales y plantados	CORANTIOQUIA
Planificación hidrológica participativa	CORANTIOQUIA
Promoción de la cultura de la legalidad ambiental	CORANTIOQUIA
Conservación y manejo sostenible del bosque, la flora y la fauna	CORANTIOQUIA
Gestión ambiental integral articulada a lo local	CORANTIOQUIA
Implementación de estrategias de producción mas limpia	CORMACARENA
Planificación y ordenamiento del recurso hídrico	CORMACARENA
Promocioón de alternativas de mercados verdes e implementacion de medidas sanitarias y fitosanitarias	CORMACARENA
Gestión productiva sostenible en el sector primario de la economía	CORNARE
Procesos de restauración, conservación y manejo de los ecosistemas boscosos de la región	CORNARE
Desarrollo de procesos de sensibilización y fortalecimiento de la cultura ambiental	CORNARE
Investigación aplicada para la mitigación de impactos ambientales	CORNARE
Participación interinstitucional para la formulación y seguimiento de planes ecoturísticos en la región	CORNARE
Conclusión, formulación e implementación del plan maestro para el Parque Arví Valles de San Nicolás	CORNARE
Gestión y apoyo al Parque Regional Ecoturístico Arví	CORNARE
Asesoría y apoyo en la armonización de la dimensión ambiental en la planificación del territorio	CORPMAG
Diagnóstico y manejo integral de cuencas y microcuencas en el departamento del Magdalena	CORPMAG
Proceso de ordenamiento pesquero en el departamento del Magdalena	CORPMAG
Implementación del esquema de pagos por servicios ambientales banco2 en el departamento del Magdalena	CORPMAG
Arborización urbana	CORPMAG
Investigación sobre la biodiversidad de especies de la SNSM	CORPMAG
Implementación del plan operativo de mercados verdes	CORPMAG
Plan de desarrollo sostenible de la Sierra Nevada de Santa Marta	CORPMAG
Fortalecer mecanismos para la identificación y promoción de sistemas productivos sostenibles y para la generación y aplicación de incentivos y estrategias de conservación de la biodiversidad en la región	CORPOAMAZONÍA
Plan de acción inversión generación hídrica	CORPOBOYACÁ
Formación de una cultura para la sostenibilidad ambiental	CORPOBOYACÁ
Gestión de la oferta hídrica	CORPOBOYACÁ
Educación ambiental y procesos participativos	CORPOBOYACÁ
Fomento a la producción agropecuaria y forestal sostenible	CORPOCALDAS
Participación ciudadana para la gestión ambiental integral	CORPOCALDAS
Planificación de la biodiversidad	CORPOCALDAS
Hacia una comunicación pública para la sostenibilidad ambiental	CORPOCALDAS
Capacitación para la conservación y uso sostenible de la biodiversidad	CORPOCALDAS
Implementación del plan de ordenación forestal del departamento del Cesar	CORPOCESAR
Implementación de acciones para la recuperación, restauración y aprovechamiento sostenible del recurso flora	CORPOCESAR
Ejecución del plan de manejo ambiental y acciones prioritarias de mitigación y mejoramiento de la Ecoregión Estratégica Complejo Cenagoso de Zapatosa y humedales menores	CORPOCESAR
Formulación e implementación de planes de ordenación y manejo de cuencas en el departamento del Cesar	CORPOCESAR
Educación ambiental para la formación social y participación comunitaria	CORPOCHIVOR
Implementación de sistemas agroalimentarios a través de prácticas productivas sostenibles y limpias, en el marco de cadenas productivas	CORPOCHIVOR
Promover la investigación aplicada en descontaminación de aguas	CORPOCHIVOR
Gestión y desarrollo forestal	CORPOGUAJIRA
Educación formal	CORPOGUAJIRA
Gestión ambiental urbana	CORPOGUAJIRA
Diagnóstico y acciones para la recuperación y conservación de ecosistemas	CORPOGUAJIRA
Aplicación y asistencia de iniciativas que generen empleo productivo ambiental	CORPOGUAVIO
Recuperación ecológica y/o reforestación en cuencas tributarias de acueductos municipales	CORPOGUAVIO
Mejoramiento de la educación y sensibilización ambientales	CORPOGUAVIO
Implantación de sistemas lúdicos de formación de valores ambientales	CORPOGUAVIO
Fomento a la participacion comunitaria	CORPOGUAVIO
Implementación de alternativas productivas sostenibles y capacitación a grupos de pescadores de la jurisdicción de Corpomojana	CORPOMOJANA
Adecuación y dotación para la investigación en fauna *crocodyllia*	CORPOMOJANA

Establecimiento de plantaciones protectoras para la restauración de la parte baja de las microcuencas de los arroyos Grande Corozal y Manzanares, jurisdicción de Corpomojana	CORPOMOJANA
Formulación y desarrollo del plan de ordenamiento forestal del área de jurisdicción de Corpomojana	CORPOMOJANA
Implementación de prácticas biológicas sostenibles con pequeños productores campesinos de los municipios de San Marcos, Caimito, San Benito Abad y Sucre, departamento de Sucre	CORPOMOJANA
Diagnóstico, manejo y ordenamiento de cuencas hidrográficas en el área de jurisdicción de Corpomojana	CORPOMOJANA
Establecimiento de sistemas de regulación y manejo de caudales (zona centro, norte, sur y sur occidente)	CORPONARIÑO
Ordenamiento, manejo y aprovechamiento sostenible de los bosques naturales de la Costa Pacífica	CORPONARIÑO
Ordenamiento y manejo integral de cuencas y microcuencas abastecedoras de acueductos de las cabeceras municipales (Mayo y Juanambu) zona norte	CORPONARIÑO
Conservación y manejo sostenible de los ecosistemas de Páramo Andino Amazónico (Bordoncillo, Patascoy, Humedal Ramsar la Cocha y páramos aledaños al róo Bobo-las piedras)	CORPONARIÑO
Educación ambiental y capacitación para el manejo y aprovechamiento forestal sostenible de los bosques naturales en los consejos comunitarios de la Costa Pacífica de Nariño	CORPONARIÑO
Implementación del programa de tasa de uso del agua y seguimiento a usuarios de concesiones y seguimiento a planes de uso eficiente y ahorro del agua	CORPONARIÑO
Fomento al aprovechamiento sostenible de bienes y servicios derivados de la biodiversidad bajo el enfoque de cadena de valor	CORPONARIÑO
Estructuración de la primera fase de la línea de investigación en biodiversidad, agua y suelo	CORPONARIÑO
Gestión para el ordenamiento y manejo de la cuenca hidrográfica del río Pamplonita	CORPONOR
Administración del sistema regional de áreas de manejo especial	CORPONOR
Implementacion del plan de manejo ambiental del area de influencia de la planta térmica	CORPONOR
Manejo sostenible de la biodiversidad y sistemas de producción sostenible	CORPONOR
Desarrollo productivo con sostenibilidad ambiental	CORPORINOQUÍA
Manejo integral de cuencas estratégicas	CORPORINOQUÍA
Seguimiento aguas subterráneas eje bananero	CORPOURABÁ
Plan de ordenación y manejo forestal Serranía de Abibe	CORPOURABÁ
Etnobotánica en caimán nuevo	CORPOURABÁ
Comunicaciones y educación ambiental	CORPOURABÁ
Ordenación, manejo, recuperación de bosques y biodiversidad	CORPOURABÁ
Ordenación de cuencas y aguas subterráneas	CORPOURABÁ
Administración de los recursos naturales y del ambiente	CORPOURABÁ
Producción limpia	CORPOURABÁ
Establecimiento proyectos piloto de agricultura urbana	CORTOLIMA
Biocomercio	CRA
Estrategia de comunicación en asuntos ambientales	CRA
Sistema de apoyo para la declaración y establecimiento de áreas protegidas a municipios, consolidación del sistema de red de reservas de la sociedad civil y promoción de corredores biológicos y ecoturísticos	CRA
Capacitación para el desarrollo sostenible y aprovechamiento forestal	CRA
Estudios de especies promisorias de fauna y flora	CRA
Capacitación a las comunidades a través de los promotores ambientales (capacitación técnica y ambiental a las comunidades indígenas)	CRA
Recuperación y aprovechamiento ecoturístico de las diferentes playas presentes en el departamento	CRA
Reforestación protectora y productora – protectora con especies nativas e introducidas en zonas de interés ambiental con sus respectivos mantenimientos	CRA
Recuperación y protección ambiental en los municipios del departamento	CRA
Estructuración de la red de promotores ambientales y actividades con las comunidades	CRA
Elaboración y publicación de estrategias pedagógicas para el conocimiento y conservación de los recursos de la biodiversidad desarrollados en el departamento	CRA
Formulación de los planes de ordenamiento y manejo de cuencas hidrográficas e inversión en las cuencas con POMCH aprobados	CRC
Repoblación, manejo de rodales y capacitación en la transformación de la guadua	CRC
Alternativas de producción agropecuaria sostenible	CRC
Gestión para la recuperación, conservación y protección de las poblaciones de flora silvestre	CRC
Fortalecimiento de las capacidades locales para la conservacion, recuperacion y uso sostenible del ecosistema manglar en el marco del plan ambiental de los manglares	CRC
Repoblación forestal de ecosistemas estratégicos	CRC

Producción limpia y mercados verdes	CRC
Alternativas de producción sostenible acorde con la calidad de suelos	CRC
Conservación, manejo y uso sostenible de la biodiversidad	CRQ
Sistemas sostenibles de producción rural	CRQ
Gotita de agua	CRQ
Pensemos en el futuro, ahorremos agua	CRQ
No me arriesgo	CRQ
El suelo un capital natural	CRQ
Implementación del plan de ordenamiento y manejo de la cuenca del Río La Vieja	CRQ
Apoyo en el mejoramiento ambiental de procesos productivos agrícolas, pecuarios y forestales	CRQ
Protección de fuentes de agua y recursos de conectividad del paisaje en la subcuenca de la quebrada Santo Domingo, municipio de Simití, sur del departamento de Bolívar	CSB
Recuperación de microcuencas de acueductos veredales y municipales	CSB
Implementación de prácticas sostenibles de producción y extracción en los sectores productivos con fines de mercados verdes en la jurisdicción de la CSB	CSB
Determinación del estado actual y formulación de planes de manejo para los páramos de la cordillera central del Valle del Cauca, jurisdicción CVC	CVC
Mejoramiento ambiental en el área del corredor de conservación Parque Nacional Natural Tatamá Serranía de los Paraguas con la participación de las organizaciones de base comunitaria y los entes territoriales de la región	CVC
Desarrollo de iniciativas de biocomercio de productos y servicios ambientales no maderables y fauna en el departamento del valle del cauca	CVC
Operación de modelos productivos agroecológicos	CVC
Proyecto piloto gestión integral participativa en la administración del agua en el río Tuluá	CVC
Restauración de suelos erosionados y caminos veredales en el corregimiento el Saladito, municipio de Cali	CVC
Formación ciudadana	CVC
Mejoramiento de prácticas agrícolas zona Pacífica	CVC
Diseño e implementación de herramientas de manejo de paisaje en el Jardín Botánico de Cali y su área de influencia	CVC
Gestión integral y provisión de servicios ambientales ecosistémicos para la conservación del recurso hídrico en cuencas estratégicas del Valle del Cauca	CVC
Recuperación y mejoramiento de los bienes y servicios ambientales que ofrecen los ecoparques de Tres Cruces y la Bandera en Santiago de Cali	CVC
Reconversión y mejoramiento de prácticas agrícolas y ganaderas	CVC
Cultura ambiental ciudadana para una Cali mundial	CVC
Recuperación paisajística y ambiental de zonas de espacio público en el barrio la Santa Cruz, municipio de Tulúa	CVC
Formulación del plan de ordenamiento y manejo ambiental de la cuenca hidrográfica del río San pedro	CVC
Construcción sendero ecológico Guabas-Madre Vieja Videles, municipio de Guacari	CVC
Mantenimiento de los ecoparques Pisamos y Bataclán y del vivero municipal en Santiago de Cali	CVC
Diseño e implementación de sistemas productivos sostenibles y servicios ambientales en áreas de interés ambiental	CVC
Recuperación ambiental de humedales urbanos en la zona sur de la ciudad de Santiago de Cali: El Limonar, Cañasgordas, El Retiro, Panamericano y La Babilla	CVC
Conservación y recuperación de los ecosistemas manglar y selva pluvial tropical a través de estrategias participativas en territorios colectivos de comunidades negras del Pacífico Vallecaucano	CVC
Recuperación paisajista y ambiental de la Acequia Chambimbal en el sector comprendido entre los barrios La Bombonera, Bello Horizonte y Las Palmitas en el municipio de Buga	CVC
Adecuación y recuperación física, ambiental y paisajística del jarillón y la franja protectora del río Cauca, desde la bocatoma de la planta de tratamiento de Puerto Mallarino hasta el inicio del asentamiento Samanes del Cauca	CVC
Manejo de especies arbóreas, parques, zonas verdes y control de hormiga arriera en la cuidad de Santiago de Cali	CVC
Adecuación y recuperación física, ambiental y paisajística de 40 parques barriales en las 22 comunas de Santiago de Cali	CVC
Desarrollo empresarial entorno a la cadena productiva de la guadua (*Guadua angustifolia*)	CVC
Elaboración y difusión de guías mineroambientales	CVC
Fortalecimiento de procesos socioeducativos de las comunidades étnicas del departamento orientados a la conservación de los recursos naturales y el ambiente	CVC

Asesoría al sector formal de la educación en el Valle del Cauca	CVC
Diseño, validación e implementación de la estrategia de educación ambiental y fortalecimiento de comunidades en procesos y proyectos de uso y manejo eficiente del agua	CVC
Manejo y conservación del recurso hídrico en el departamento de Córdoba	CVS
Educación ambiental como base estructural de la política regional y gobernanza	CVS
