# Supplementary material for: Integrating Ecosystem Services in Nature Conservation for Colombia
Source: Environ Manage. 2020 May 28;66(2):149–61. doi: 10.1007/s00267-020-01301-9 (PMC7320067; doi:10.1007/s00267-020-01301-9)
Supplement: Supplementary file 3 — Supplementary Material 3 [file 267_2020_1301_MOESM3_ESM.pdf]

Appendix 3 Coding framework, labels and description of variables used in the descriptive and MCA/HCA analysis of the projects.

N.	Variable and items	Code	Label	Description	Analysis
1	**ES consideration [a]**				Descriptive
	Explicit in the goals of the project	1	*explicit/goal*	Specific use of the term "ecosystem services/environmental services" within the goals. Clear mention that nature provide s to humankind	
	Implicit in the goals of the project	2	*implicit/goal*	Notion that nature provides benefits to humankind within the goals. No specific term used within the goals	
	Explicit throughout the body of the project	3	*explicit/body*	Specific use of the term "ecosystem services/ environmental services" within the body of the project. Clear mention that nature provide benefits to humankind within the body of the project	
	Implicit throughout the body of the project	4	*implicit/body*	Clear notion that nature provides benefits to humankind along the project. No specific term used along the project	
2	**ES section [b] ***				MCA/HCA Descriptive
	Provisioning	1	*provisioning*	All nutritional, material and energetic outputs from living systems	
	Regulating & Maintenance	2	*regulating*	Covers all the ways in which living organisms can mediate or moderate the ambient environment that affects human performance.	
	Cultural	3	*cultural*	Covers all the non-material, and normally non-consumptive, outputs of ecosystems that affect physical and mental states of people. Education was a frecuently cultural ES.	
3	**Purpose of CEPA actions [c]**				MCA/HCA Descriptive
	Without CEPA actions	1	*without CEPA actions*	Projects that not included CEPA actions	
	CEPA as a mean (a strategy for conservation)	2	*CEPA as a mean*	Project defines specific conservation targets that it ultimately wants to influence project goals are focused on the project potential contribution to resolving environmental issues. CEPA actions are a component of multidisciplinary conservation efforts	
	CEPA as an end (as the main goal)	3	*CEPA as an end*	Project does not target tangible conservation objectives, but it is believed that the implementation of any CEPA actions indirectly supports conservation project goals focus on the educational component rather than in the environmental component.	
4	**E. communication (EC) [d]**				MCA/HCA
	Without EC	1	*without EE*	Projects that not included EC	
	EC	2	*communication*	The process of developing and exchanging messages among different social agents aimed at promoting environmentally friendly and sustainable knowledge, attitudes, and behaviors.	
5	**E. education (EE) [d]**				MCA/HCA
	Without EE	1	*without EE*	Projects that not included EE	
	EE	2	*education*	The learning process through which knowledge of conservation principles and problems increase and attitudes and behaviors that show concern for the environment are promoted through an appropriate pedagogical process.	
6	**E. participation (EP) [d]**				MCA/HCA
	Without EP	1	*without EP*	Projects that not included EP	
	EP	2	*participation*	The range of strategies aimed at building the capacity of citizens to make decisions related to conservation. Strategies include training, policy development, and collaborative management and involve the educator and the learner working together to define the goals and methods of interventions.	
7	**Integration of type CEPA actions [c]**				MCA/HCA
	None integration	1	*none integration*	Projects that not included CEPA actions	
	Low integration	2	*low*	Only one type CEPA was present in the project	
	Medium integration	3	*medium*	Two types of CEPA were present in the project	

	High integration	4	*high*	All CEPA (EC, EE, EP) were present in the project	
8	**Main conservation actions** [e]				MCA/HCA Descriptive
	Land/water management	1	*land/water management*	Actions directed at conserving or restoring sites, habitats and the wider environment	
	Species management	2	*sps. management*	Actions directed at managing or restoring species, focused on the species of concern itself	
	Education and awareness	3	*education & awareness*	Actions directed at people to improve environmental understanding and skills, and influence behavior	
	Sustainable livelihood	*4*	*livelihood*	Actions to use the nature for economic and sustainable activities	
9	**Main stakeholders** [f]				MCA/HCA Descriptive
	Without stakeholder	1	*without stakehold.*	Projects that not included any actions engaging social actors	
	Local communities	2	*local communities*	General social group targeted in the project (mentioned commonly as community, participants, beneficiary families, local people, inhabitants, community groups, indigenous/afro communities)	
	Small-scale producers	3	*small-scale*	Small producers, farmers, and small associations (e.g. ecotourism, artisans)	
	School children	4	*school children*	Students and children	
	Environmental government staff & Decision makers	5	*env. gov. Staff & decision makers*	RAC staff, Natural Parks and protected areas committees staff, mayors, basin councils, community council of the indigenous, indigenous cabildo, municipal authorities	
	Environmental leaders & groups	6	*env.people*	Environmental promoters, environmental education committees, Communal Action board, Environmental National Police.	
10	**Women** [c,f]				Descriptive
	Without women	1	*without women*	Projects that not included women	
	Women	2	*women*	Women was targeted by the project (not necessarily as the main actor)	
11	**Unrepresented population** [f]				Descriptive
	Without unrepresented population	1	*without unrepres.*	Projects that not included indigenous and Afro-descendent communities	
	Indigenous and Afro communities	2	*indigen/afro*	Indigenous and Afro-descendent communities were targeted by the project (not necessarily as the main stakeholder)	
12	**Spatial scale** [c]				MCA/HCA
	Regional	1	*regional*	> 4 municipalities, frequently covered all RAC	
	Local	2	*local*	limiter to four minicipalitie	
13	**Budget** [g]				MCA/HCA Descriptive
	Unspecified	1	*unspecified*	Some projects there are not budget in one or any year	
	Low	2	*low budget*	<150000 US	
	Medium	3	*medium budget*	150.000 to 500.000 US	
	High	4	*high budget*	>500.000 US	
14	**Duration** [h]				MCA/HCA Descriptive
	One year	1	*one year*	Duration of the project: one year	
	2-4 years	2	*2-4 years*	Duration of the project: 2-4 years	
	>4 years	3	*>4 years*	Duration of the project: > 4 years	
15	**General goal (GG)** [g]				Descriptive
	Without GG	1	*without GG*	Projects without general goal	
	GG	2	*general goal*	Description of general goal in the projects	
16	**Specific goals** [g]				Descriptive
	Without SG	1	*without SG*	Projects without specifics goals	
	SG	2	*specific goal*	Description of specific goals in the projects	
17	**Triennial Action Plans** [h]				Descriptive
	TAP 1 (2004-2006)	1	*TAP1*	The project was developed between 2004-2006	
	TAP 2 (2007-2011)	2	*TAP2*	The project was developed between 2007-2011	
	TAP 3 (2012-2015)	3	*TAP3*	The project was developed between 2012-2015	
18	**Continuity of the project in the TAPs** [h]				Descriptive
	One TAP	1	*oneTAP*	The project lasted only one TAP	
	Two TAP	2	*twoTAP*	The project lasted two TAPs	

19	**Number of projects by RAC**					Descriptive
		<20	1	<20	<20 projects	
		20-40	2	20-40	20-40 projects	
		41-60	3	41-60	41-60 projects	
		61-80	4	61-80	61-80 projects	
		81-100	5	81-100	81-100 projects	
		101-150	6	101-150	101-150 projects	
		>150	7	>150	>150 projects	

[a] Based on Goldman and Tallis 2009; Hansen et al, 2015. Nordin et al. 2017

[b] Based on Haines-Young R, Potschin M (2018)

[c] Based on Jiménez et al. 2014 and 2015

[d] Based on Jiménez et al. 2014 and 2015; Hesselink et al. 2007

[e] Salafsky et al., 2008

[f] Based on United Nations, 1992

[g] Based on Goldman & Tallis, 2009

[h] Emergent information

* for specific explanation see Table 1
