# Supplementary material for: Integrating Ecosystem Services in Nature Conservation for Colombia
Source: Environ Manage. 2020 May 28;66(2):149–61. doi: 10.1007/s00267-020-01301-9 (PMC7320067; doi:10.1007/s00267-020-01301-9)
Supplement: Supplementary file 4 — Supplementary Material 4 [file 267_2020_1301_MOESM4_ESM.pdf]

Appendix 4 Factor scored derived from Multiple Correspondence Analysis (MCA) to know social actions in the conservation projects. Bold scores have statistical significance (p <0.05)

Label of variables	Factor scores	
	F1	F2
provisioning	-0,083	0,186
regulating	0,111	**0,219**
cultural	-0,049	**-0,396**
without CEPA actions	**1,207**	0,068
CEPA as a strategy	**-0,161**	**0,167**
pure CEPA	-0,267	**-0,680**
without env. communication	**0,400**	0,040
env. communication	**-0,274**	-0,028
without env. education	**0,537**	-0,053
env. Education	**-0,251**	0,025
without env. participation	**0,203**	**-0,130**
env. participation	**-0,259**	**0,166**
management	0,133	**0,166**
sps. management	0,203	-0,006
CEPA actions	-0,257	**-0,667**
livelihood	-0,106	0,152
any integration	**1,207**	0,068
low	0,084	-0,186
medium	**-0,214**	0,000
high	**-0,431**	0,167
regional	-0,066	-0,015
local	0,212	0,049
unespecified	0,305	-0,088
low budget	0,081	-0,136
medium budget	-0,151	0,053
high budget	-0,134	0,223
one year	**0,267**	-0,179
2-4 years	-0,134	0,056
>4 years	-0,184	0,182
without stakehold.	**1,207**	0,068
local communities	-0,153	0,003
small-scale	-0,260	0,184
school children	-0,277	-0,377
env. gov. Staff & decision makers	-0,086	0,193
pro-env. people	-0,192	-0,229
**MCA values**		
Eigenvalues	0,101	0,036
Adjusted inertia (%)	49,642	17,673
Cumulative inertia (%)	49,642	67,314
