# Supplementary material for: Integrating Ecosystem Services in Nature Conservation for Colombia
Source: Environ Manage. 2020 May 28;66(2):149–61. doi: 10.1007/s00267-020-01301-9 (PMC7320067; doi:10.1007/s00267-020-01301-9)
Supplement: Supplementary file 5 — Supplementary Material 5 [file 267_2020_1301_MOESM5_ESM.pdf]

Appendix 5 Number of conservation projects in each of the 4 groups of CEPA projects identified by the hierarchical cluster analysis (HCA).

Variable and items [a]	Environmental management without society engagement *(n=24)*	Environmental awareness *(n=32)*	Integrated participation for fostering sustainable livelihoods *(n=49)*	Small conservation actions with local communities *(n=77)*	*Chi-square test* [b]	*df*
**ES category**					12.59**	6
provisioning	6	0*	22*	24		
regulating	12	0*	25*	30		
cultural	6	32*	2*	23		
**Purpose of CEPA actions**					12.59**	6
without CEPA actions	24*	0*	0*	0*		
CEPA as a strategy	0*	0*	49*	76*		
pure CEPA	0*	32*	0*	1*		
**E. communication**					7.81**	3
without EE	24*	5*	9*	36		
EC	0*	27*	40*	41		
**E. education**					7.81**	3
without EE	24*	7	0*	27		
EE	0*	25	49*	50		
**E. participation**					7.81**	3
without EP	24*	21	5*	52*		
EP	0*	11	44*	25*		
**Integration of type CEPA actions**					16.91**	9
none integration	24*	0*	0*	0*		
low	0*	11	0*	38*		
medium	0*	11	14	39*		
high	0*	10	35*	0*		
**Main conservation action**					16.91**	9
land/water management	18*	0*	29	46		
sps. management	1	0*	0*	6*		
education&awarenes	0*	32*	0*	2*		
livelihood	5	0*	20*	23		
**Main stakeholders**					24.96**	15
without stakehold.	24*	0*	0*	0*		
local communities	0*	13	22	39*		
small-scale	0*	1*	17*	14		
school children	0*	5*	2	2		
env. gov. Staff & decision makers	0	1	5	7		
env.people	0	12*	3*	15		
**Spatial scale**					7.81**	3
regional	16	30*	42	51*		
local	8	2*	7*	26*		
**Budget**					16.91**	9
unspecified	6	4	7	8		
low budget	9	16	9*	40*		
medium budget	4	8	14	18		
high budget	5	4	23*	11*		
**Duration**					12.59**	6
one year	14*	15	3*	34		
2-4 years	7	14	28*	25*		
>4 years	3	3*	18*	18		

[a] Only variables/items whose p-value for chi-squares test was less than 0.1 are compiled in this table

[b] Significance value: *p<0.05; **p<0.001
